# Feasibility of Implementing Infant Home Visiting in a Central Australian Aboriginal Community

**DOI:** 10.1007/s11121-018-0930-5

**Published:** 2018-07-28

**Authors:** Ha Nguyen, Dorota Zarnowiecki, Leonie Segal, Debra Gent, Bronwyn Silver, John Boffa

**Affiliations:** 1 Health Economics & Social Policy Group, Centre for Population Health Research, University of South Australia, Adelaide, South Australia Australia; 2 Centre for Population Health Research, South Australia Health & Medical Research Institute (SAHMRI), GPO Box 2471, Adelaide, SA 5001 Australia; 3 Central Australian Aboriginal Congress Aboriginal Corporation, Alice Springs, Northern Territory Australia

**Keywords:** Infant home visiting programme, Attrition, Retention, Aboriginal remote

## Abstract

The Australian Nurse-Family-Partnership Program, an adaption of the Olds’ Nurse-Family-Partnership (NFP), commenced in Alice Springs in 2009 (Central Australia FPP), aiming to improve the health and social outcomes of Aboriginal mothers and infants. This study explores the feasibility of NFP implementation in a remote Australian Aboriginal community. Feasibility was defined by programme uptake by eligible women, retention in the programme, actual vs. scheduled visits and extent of programme content delivery. Programme uptake was established from pregnancy data in the patient Clinical Information System and programme referrals to December 31, 2015. Rates of withdrawal, retention and content delivery were derived from FPP data and compared with published NFP data. Modified Poisson regression was used to identify client characteristics associated with retention beyond the child’s first birthday. There were 469 valid referrals (43% of eligible pregnancies) and 299 women with at least one completed home visit by December 31, 2015. Of these, 41% completed the programme to the child’s second birthday and 53% beyond the child’s first birthday. Dominant reasons for leaving were “moved out of service area” (35%) and “declined further participation” (35%). There was a statistically significant positive association for programme retention with later gestational age at referral (RR = 1.27, *p* value = 0.03). A high proportion (75%) of scheduled visits was achieved and high delivery of programme content (80%). Central Australia FPP is the first implementation of the NFP model in a remote Aboriginal community. This study found that it can be implemented successfully in this setting. Outcome evaluation is needed to test achievement of hypothesised benefits.

## Background

In Australia, Aboriginal^1^ children are more likely to experience poorer health and development outcomes than other Australian children. Despite a massive reduction in infant mortality rates since the 1970s with the growth of the Aboriginal community-controlled health sector (Condon et al. 2001), birth outcomes are still poorer for Aboriginal Australians than for non-Aboriginal Australians. The infant mortality rate and risk of low birth weight (2011–2013) in Aboriginal children are twice that of other Australian infants (Australian Indigenous HealthInfoNet 2015). Aboriginal children are considerably more likely to be exposed to domestic violence (Twizeyemariya et al. 2017) or child abuse and neglect. In 2015–2016, 43.6/1000 Aboriginal children had a substantiated child protection concern compared with 6.4/1000 non-indigenous children (Productivity Commission 2016). The Australian Early Development Census 2012 (2014), which covers 96.5% of children in their first year of school, found a greater proportion of developmentally vulnerable children among Aboriginal than among other Australian children across all five domains of early childhood development (physical health and well-being, social competence, emotional maturity, language and cognitive skills, and communication skills and general knowledge). The largest difference in vulnerability was in language and cognitive skills (41.9% among Aboriginal children and 16.0% among other Australian children).

Poor health and social outcomes in First Nation’s Peoples are universally observed, reflecting histories of colonisation, dispossession, child removal, racism and associated low socio-economic status (Bryant 2009; Productivity Commission 2014). The challenge is to find effective programmes that make a difference from early in a child’s life, and which address the broader social and economic determinants.

Home-based visiting during the early years of life has emerged as a promising strategy to improve health and developmental outcomes for children, particularly those in more socio-economically disadvantaged communities (Kitzman 2007). These programmes are characterised by a home visitor (a nurse, social worker, trained paraprofessional or layperson) who provides a range of services that may include parenting education, case management, social supports, and advocacy for mothers and their infants, delivered primarily in the client’s home. Many, but not all, programmes commence during pregnancy and most continue until the child is between 6 months and 2 years of age (Segal et al. 2012; Sweet and Appelbaum 2004). The aim is to intervene early to support new parents in establishing a nurturing parenting style, to enhance the quality of the attachment relationship and ensure the physical, nutritional and emotional nourishment needed by the developing infant (Raikes et al. 2006; Shonkoff et al. 2009). Offering a home-based visiting service is designed to increase uptake by vulnerable families who might be less likely to attend centre-based appointments. Establishing a warm and trusting relationship between the home visitor and the client is important in facilitating programme acceptance.

### The Australian Nurse-Family-Partnership Program in Central Australia

In March 2009, an Aboriginal community-controlled health service in Alice Springs, Central Australia, commenced delivery of the Australian Nurse-Family-Partnership Program (ANFPP). This was part of an initiative of the Australian Government to implement a culturally adapted Olds’ Nurse-Family-Partnership Program (NFP) (Olds et al. 1999). The aims of the ANFPP are, through a series of scheduled home visits from pregnancy to the child’s second birthday, to improve pregnancy outcomes (e.g. premature birth/low birth weight), enhance child health and development (e.g. reduce child protection system involvement) and assist mothers in their own development.

The Olds’ NFP is one of a small number of home-visiting programmes with evidence of a reduction in child abuse and neglect (Olds et al. 1997). During home visits, clients are provided advice across six programme areas: personal health (health practices, nutrition/exercise, substance use and mental health); environmental health (home, community, local area, work and school); life course development (family planning, education and livelihood); maternal role (physical, behavioural and emotional care of infant); family and friends (personal networking relationships, assistance with child care); and health and human services (about community services available). Implementation of the ANFPP was proposed as a community-level early investment in the future of Aboriginal children.

The major adaptation to the NFP for delivery in the Central Australian Aboriginal context was the inclusion of Aboriginal community workers (ACWs) to deliver the programme alongside the trained nurse home visitors (NHVs). The primary role of the ACWs was the provision of cultural brokerage, advice and support to the FPP team regarding cultural issues and community conditions and to establish and maintain cultural safety for clients and their families. The ACW accompanies the NHV on initial visits, introducing the NHV to the client and encouraging family members to be supportive of the client’s efforts to participate in the programme. The ACW manages the informed consent process and assists in maintaining client contact, which is particularly important in a highly mobile population. The ACWs participate in cultural and community events to raise awareness of the programme with elders, clients’ extended families and other community members.

A further adaption was to extend programme eligibility to multiparous women in order to maximise programme reach, given the desire to improve maternal and child outcomes across the community. Programme materials were largely unchanged, thus maintaining fidelity with core content of the NFP as an “evidence-based” programme, (https://www.anfpp.com.au/). The Central Australia FPP is delivered alongside a universal maternal and child health service.

### Aims of the Study

This paper aims to explore the feasibility of implementing the ANFPP model in Central Australia when delivered by an Aboriginal community-controlled organisation that provides comprehensive primary health care services to the Aboriginal population in and around Alice Springs.

The broader context was to determine whether a modified NFP could successfully be implemented in a highly mobile and disadvantaged population of First Nations People living in a remote setting in Australia.

## Methods

### Study Sample

The study sample includes all Aboriginal women having a pregnancy outcome recorded in the Central Australia Aboriginal Health Service’s Client Information System (CIS) between March 1, 2009, and December 31, 2015. Women were considered eligible for the programme if they (i) were pregnant with an Aboriginal baby; (ii) were referred to the Central Australia FPP at less than 28 weeks-gestation; (iii) lived (recorded locality) in the programme catchment area of Alice Springs or within 100 km when 13 to 28 weeks-gestation; and (iv) had not previously been enrolled in the Central Australia FPP. Women who had an early (less than 13 weeks gestation) miscarriage, termination or abortion were excluded from our sample of eligible women.

### Data

De-identified data for the study were extracted from the CIS, including the residential location of potentially eligible women, all pregnancy outcomes between March 1, 2009, and December 31, 2015 and the Central Australia FPP programme data. For clients who were “still active” as at December 31, 2015, their participation status was updated on March 22, 2018. Residential location when 13 to 28 weeks pregnant was used to determine eligibility for the programme. All residential addresses recorded between March 1, 2007, and December 31, 2015, were used to measure housing stability, as an indicator of vulnerability. To ensure confidentiality of client data, coding of localities (as “in” or “out” of the programme catchment area) and flagging of house moves (with an associated date) was completed by staff of the Central Australia Aboriginal Health Service.

To estimate coverage or reach of the programme since its inception in March 2009, pregnancy outcome data were extracted to identify all pregnancies apparently eligible for referral to the Central Australia FPP.

Central Australia FPP data were derived from:

- *Referral form*, which included basic information about referral source, outcome (whether referred women chose to participate in the programme), gestational age at referral and the number of previous live births
- *Demographic details form*, administered within the first four visits, covering client socio-economic characteristics—such as living arrangement, education level, employment status and income
- *Home visit encounter form*, completed on each “home” visit and covering, for example, the location of the visit, the level of client engagement and the time spent on each of the six programme domains
- *Client change of status form*, completed when a client reached a programme milestone (such as birth of baby, child’s second birthday) or when there was a change in participation status (such as declining further participation, miscarriage/infant death, moving out of service area) (Australian Nurse-Family Partnership 2011).

### Measures

*Programme coverage* or uptake was estimated as the total number of pregnant women who enrolled in the FPP and had at least one home visit by December 31, 2015, relative to the total number of eligible pregnancies.

*Programme participation status* was categorised into one of four possible groups:

- *Completed*, women who remained in the programme until the child’s second birthday
- *Left—pregnancy*, women who left the programme prior to the child’s birth
- *Left—infancy*, women who left the programme after the child’s birth but prior to the child’s first birthday
- *Left—toddlerhood*, women who left the programme after the child’s first birthday but prior to the child’s second birthday

Participation status was identified through the *Client change of status form*, or, if 180 days had lapsed since the last home visit, the client was identified as having left the programme. These criteria align with those used by O’Brien and colleagues in their study of attrition in the NFP in the USA (O'Brien et al. 2012).

*Programme retention* and attrition were estimated to the child’s first birthday and to programme completion (child’s second birthday) based on client participation status as recorded in the CIS by March 22, 2018.

*Addressable attrition* was defined to include the four categories: (i) declined further participation, (ii) not able to be located, (iii) excessive missed appointments and (iv) more than 180 days since their “last” visit (O'Brien et al. 2012).

*Intensity of visits* was estimated as the ratio of the number of completed visits that clients received relative to the intended number of home visits defined by the FPP programme delivery protocol. The programme was based on weekly visits for the first 4 weeks after enrolment, then fortnightly visits until the child’s birth, weekly visits for the first 6 weeks after the child’s birth, fortnightly visits until 21 months and monthly visits until the child turns two years of age (https://www.anfpp.com.au/). This equates to 29 visits during the first year of life and 23 visits during the second year; expected visits during pregnancy depend on the gestational age when the client starts the programme and gestational age at birth/birth outcome. The number of intended visits during pregnancy was thus estimated specifically for each client, to reflect their gestational age on commencement into the programme and at birth outcome.

The US NFP model provided the benchmark for comparing programme retention and other implementation outcomes (Olds et al. 1999; O'Brien et al. 2012), noting positive maternal and child outcomes reported for this programme.

### Analyses

Descriptive statistics (count and proportion) were used to describe the socio-demographic characteristics of all eligible women for the period March 18, 2009, to December 31, 2015. Analysis of variance and chi-square tests were used to compare characteristics between groups defined by referral and acceptance status. More detailed socio-demographic characteristics were described for all FPP women who had completed a demographic form.

Analyses of factors associated with the likelihood of staying with the programme beyond the child’s first birthday were performed for clients with at least one completed home visit. Modified Poisson regression was used to estimate the risk ratio (RR) of retention in the programme beyond the child’s first birthday. RR is used as the more intuitive measure of association between exposure and outcome and as such is easier to interpret than odds ratios (OR) (often estimated in logistic regression) (Viera 2008). Modified Poisson regression is applied to binomial data using a robust error variance and is found to estimate RR consistently and efficiently (McNutt et al. 2003; Zou 2004). We examined univariate relationships between women’s socio-demographic characteristics and the likelihood of staying in the programme beyond the child’s first birthday. All analyses were performed using STATA 12 (https://www.stata.com).

## Results

### Programme Uptake and Coverage of the Eligible Population

**Fig. 1:**
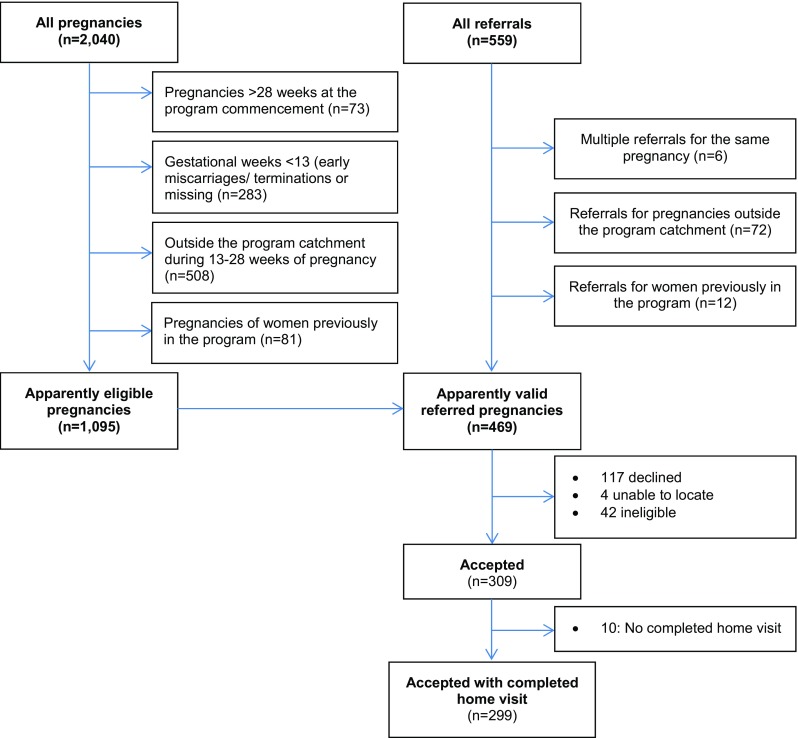
Pregnancies eligible for the programme and valid referrals

A total of 2040 pregnancies were recorded in the CIS between March 1, 2009, and December 31, 2015, of which 1095 were identified as eligible for the Central Australia FPP programme. Reasons for exclusion are summarised in Fig. 1. The 469 apparently valid referrals for the period between March 18, 2009, and December 31, 2015, represent nearly 43% of the 1095 eligible pregnancies. A further adjustment was necessary, as 42 referrals were found to be ineligible by the Central Australia FPP team (largely reflecting change in locality details), leaving 427 eligible referrals. The 309 women who accepted the programme represented 72% of eligible referrals.

While just under half of all eligible women were referred into the programme, a non-referral does not necessarily mean that a woman was unaware of the programme. She may have been offered a referral but showed no interest.

**Fig. 2:**
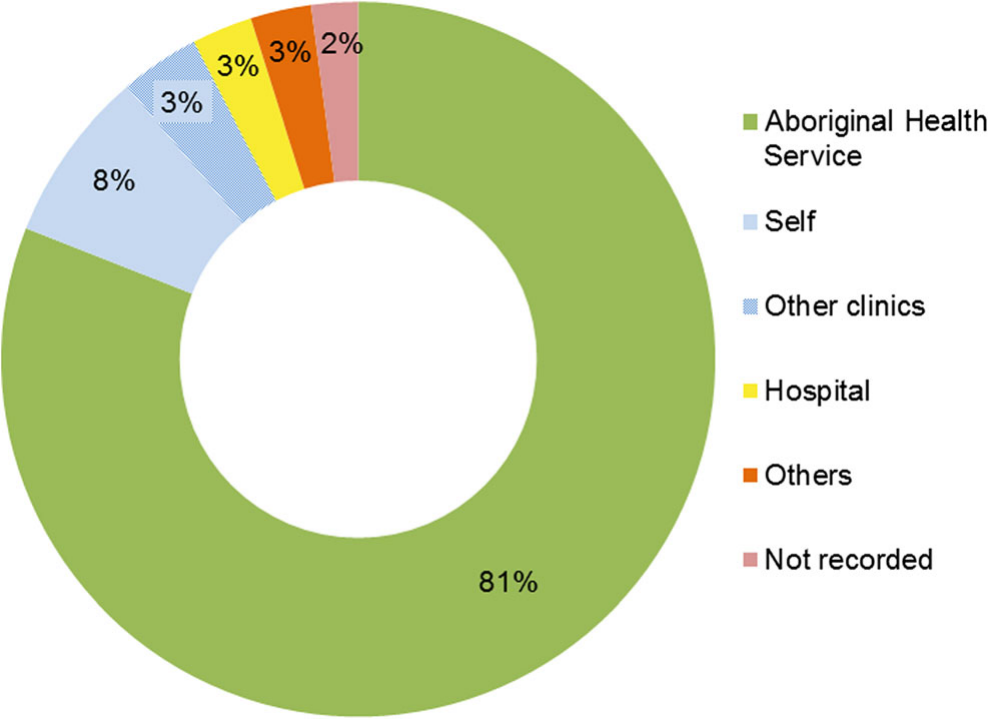
Sources of referrals to the Central Australian FPP

Most referrals to the Central Australia FPP (81%) came from other programmes or services within the Central Australia Aboriginal health service (mainly from the women’s health programme and the general clinic). Other referral sources included self-referral, other health care providers, private clinics and the hospital (Fig. 2).

### Socioeconomic Characteristics of Eligible Women and FPP Clients

Key characteristics of eligible women are described in Table 1 by four groupings: group 1—eligible but not referred; group 2—eligible, referred and declined; group 3—eligible, referred and not locatable, or identified as ineligible by FPP staff; group 4—eligible, referred and accepted the programme. There are significant differences in socio-demographic characteristics between the four groups. The mean age of women in group 4 (in the programme) was younger than women in group 1 (not referred) and group 2 (declined participation) (*p* value < 0.001), reflecting a higher proportion of women in group 4 aged less than 18 years and fewer over 30 years of age (*p* value < 0.001). The proportion of women in their first pregnancy was highest in group 4, more than twice that in group 2 (*p* value < 0.001). The proportion of women in group 4 with three or more children is lower than in other groups, but still sizable at over one third of women. The number and rate of house moves, an indicator of housing instability and vulnerability, were statistically significantly higher in women in FPP women (group 4) than women not referred (group 1) (*p* value < 0.001). (House moves were measured over a mean observeation period of 5 years from March 1 2017 to date of referral or 13 weeks gestation.

**Table 1:** Comparison of groups by referral and acceptance status

	Groups by participation status								
	Group 1 (*n* = 626)		Group 2 (*n* = 114)		Group 3 (*n* = 46)		Group 4 (*n* = 309)		*p* value*
	*n*	%	*n*	%	*n*	%	*n*	%	
Age (years)									
Mean	25.6		26.1		24.9		23.1		< 0.001
< 18	59	9.4	5	4.4	7	15.2	45	14.7	< 0.001
18–20	111	17.7	21	18.4	6	13.0	75	24.4	
21–25	153	24.4	32	28.1	10	21.7	99	32.2	
26–30	157	25.1	24	21.1	15	32.6	57	18.6	
> 30	146	23.3	32	28.1	8	17.4	31	10.1	
Pregnancy									
First	163	26.2	19	16.7	15	32.6	130	43.5	< 0.001
Second	113	18.2	26	22.8	5	10.9	65	21.7	
Third or more	345	55.6	69	60.5	26	56.5	104	34.8	
Number of house moves between March 1, 2007, and the 13-week gestation or referral to the Congress FPP									
Mean time period (years)	4.88		5.27		4.88		5.26		0.018
Mean moves per year	0.504		0.641		0.703		0.680		<0.001
≥ 5 moves (*n*, %)	100	16.0	29	25.4	11	23.4	78	25.2	0.003
≥ 1 move/year (*n*, %)	96	15.3	21	18.4	12	25.5	65	21.0	0.071

Group 1 = eligible but not referred; group 2 = eligible, referred and declined; group 3 = eligible, referred and not locatable or identified as ineligible by the FPP staff; group 4 = eligible, referred and accepted

*p values of analysis of variance for continuous variables and chi-square test for categorical variables

Socio-economic characteristics of women who commenced the Central Australia FPP and completed the *Demographic Details Form* (89%) are presented in Table 2. The most common living arrangement was “living with husband/partner and others but not own mother” (34.2%), (often including the partner’s mother). Only 14.7% of women lived with their “husband/partner only” and 13.9% lived with their “own mother only”.

**Table 2:** Characteristics of FPP women who completed the demographic form (*n* = 266)

Characteristics	FPP	
	*n*	%
Living arrangement		
Husband/partner and others, but not own mother	91	34.2
Other adults, not own mother or husband/partner	42	15.8
Husband/partner only	39	14.7
Own mother only	37	13.9
Own mother and husband/partner	34	12.8
Alone	7	2.6
Live in group home/shelter/homeless	8	3.0
Not recorded	8	3.0
Highest year of school		
Year 11–12 or equivalent	92	34.6
Year 9–10 or equivalent	134	50.4
Year 6–8 or below	30	11.3
Not recorded	10	3.8
Professional qualification		
Bachelor/diploma	10	3.8
Vocational/technical/trade	56	21.1
Some non-school training—not completed	22	8.3
None	167	62.8
Not recorded	11	4.1
Currently working		
No	173	65.0
Full time	29	10.9
Part time	16	6.0
Not recorded	48	18.0
Estimated personal income		
≤ $500 pw	199	74.8
$500–$999 pw	40	15.0
≥ $1000 pw	11	4.1
Not recorded	16	6.0
Main source of income		
Government or other welfare benefits	168	62.8
Husband/partner	25	9.4
Working (wages)	38	14.3
Other	23	8.6
Not recorded	12	4.5

Most of the women had 10 years or less of schooling (61.7%). Less than 25% of the women had completed some type of non-school qualification. Only 16.9% of women were working either full- or part-time at the time of programme intake. The majority (74.8%) of women were on very low incomes, with the most common source of income a government welfare payment.

### Retention and Attrition in the Central Australia FPP

**Fig. 3:**
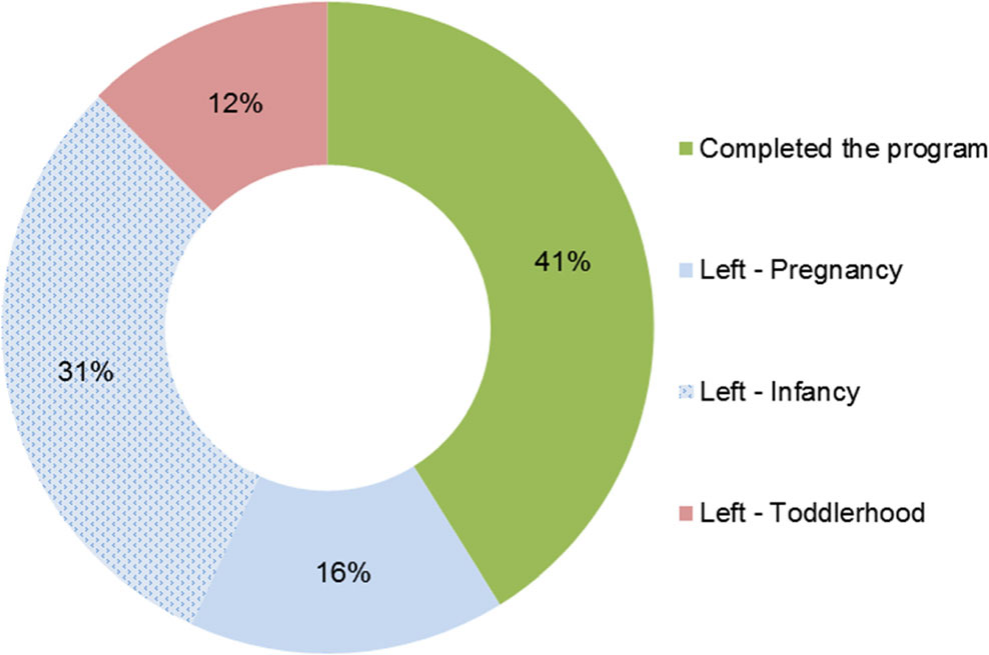
Client’s participation status as at March 31, 2018

Participation status of the 299 clients who completed at least one home visit is presented in Fig. 3. By March 22, 2018, when all children in the cohort had reached at least 2 years of age, 123 (41%) clients had completed the full programme from pregnancy to the child’s second birthday, 47 (16%) had left before the child’s birth, 92 (31%) left after the child was born but before turning 1 year old and 37 (12%) left when the child was between 1 and 2 years of age. The programme retained 53% of women to the child’s first birthday (and beyond) and 41% to the child’s second birthday.

Reasons for leaving the programme prior to the child’s first birthday are summarised in Table 3 for women in the Central Australia FPP together with comparative data for the US NFP. For the Central Australia FPP, most common reasons for leaving the programme before the child’s first birthday were “client declining further participation” or “moving out of the service area”. Only 2.2% left the programme as “unable to locate”, compared with 13.9% in the US NFP. Less than 60% of those leaving the Central Australia FPP were classified as addressable, compared with nearly 80% for the US NFP. Further exploration of reasons for clients declining further participation found the largest group “had received what they needed from the program”, with a few “returning to work”, one citing “pressure from family” and another “refusing new nurse visitor”.

**Table 3:** Reasons for leaving the program before the child’s first birthday

Reasons for leaving the program	FPP		US NFP^	
	*n*	%	*n*	%
Non-addressable attrition				
Miscarriage/foetal or infant death	6	4.3	285	5.6
Child not in family custody/parental right terminated	4	2.9	97	1.9
Moved out of the service area	49	35.3	667	13.0
Sub-total	59	42.4	1049	20.4
Addressable attrition				
Declined further participation*	49	35.3	1508	29.4
Unable to locate	3	2.2	713	13.9
Excessive missed appointments/attempted visits	28	20.1	1864	36.3
Subtotal	80	57.6	4085	79.6
Total	139	100	5134	100

^O’Brien et al., reports on NFP delivery across 66 communities in the USA from July 1, 1999, to December 31, 2001 (O'Brien et al. 2012)

*Reasons: 13: received what they need, 2: returning to work, 1: receiving services from another program, 1: pressure from family, 1: not wanting to change to new NHV, 1: incarcerated/institutionalised, 30: not specified

The analyses of client characteristics associated with likelihood of retention beyond the child’s first birthday are reported in Table 4. The focus is on whether more vulnerable women were more or less likely to be retained in the programme. Programme retention rates varied between 40 and 71% for women with different characteristics. Among the groups that might be considered particularly vulnerable, retention to at least the child’s first birthday was higher than the average (53%). For example, for women who were living in a shelter or were homeless, 60% retained; for women with likely depression, 71% retained; for women “afraid of a current or previous partner”, 65% retained; and for women with three or more children, 67% retained. The analyses of risk ratios comparing retention rates between groups found a statistically significant difference for gestational age at referral. Women referred later, a more vulnerable group, were more likely to be retained beyond the child’s first birthday (RR 1.27, *p* = 0.03).

**Table 4:** Characteristics at referral and likelihood of retention in FPP beyond child’s first birthday

Characteristics at referral	N*	(%) Retention beyond child 1st birthday^#^	Univariate	
			RR	*p* value (95% CI)
Data from referral form				
Gestational age at referral (weeks)				
19 weeks or less	190	(47%)	1.00	
20 weeks or more	87	(61%)	1.27	0.03 (1.02–1.56)
Age group (years)				
19 years or less	98	(49%)	1.00	
20 years or more	187	(53%)	1.11	0.40 (0.88–1.40)
Number of previous live births—all				
1	68	(40%)	0.74	0.06 (0.54–1.01)
2	26	(54%)	0.98	0.91 (0.69–1.40)
3 or more	36	(67%)	1.20	0.17 (0.92–1.56)
Data from demographic form				
Living arrangement				
Own mother only	36	(47%)	1.00	
Own mother and husband/partner	33	(52%)	1.09	0.72 (0.69–1.72)
Husband/partner only	35	(57%)	1.26	0.27 (0.84–1.91)
Husband/partner and other adults	86	(59%)	1.24	0.25 (0.86–1.80)
Other adults, but not own mother nor husband/partner	40	(58%)	1.22	0.34 (0.811.85)
Alone/shelter/homeless	14	(57%)	1.23	0.44 (0.732.10)
Number living in household				
5 or less	132	(56%)	1.00	
6 or more	102	(58%)	1.05	0.68 (0.851.29)
Highest year of school				
Year 11–12	83	(58%)	1.00	
Year 9–10	132	(55%)	0.90	0.37 (0.73–1.13)
Year 8 or lower	28	(54%)	0.86	0.43 (0.591.25)
Non-school qualification				
Bachelor/diploma/vocational	64	(64%)	1.00	
University/TAFE/etc. not completed	19	(47%)	0.84	0.41 (0.55–1.28)
None	158	(53%)	0.85	0.14 (0.68–1.06)
Currently working				
Not working	164	(56%)	1.00	
Full time	25	(56%)	1.06	0.70 (0.78–1.45)
Part time	16	(44%)	0.75	0.32 (0.42–1.33)
Estimated personal income				
$500 or less per week	188	(56%)	1.00	
More than $500 per week	48	(54%)	0.98	0.86 (0.75–1.27)
Main source of income				
Centrelink or other benefits	161	(58%)	1.00	
Other (e.g. from partner, wages)	80	(50%)	0.92	0.49 (0.73–1.16)
Data from health assessment form				
Edinburgh Postnatal Depression Score				
0–9	75	(69%)	1.00	
10–12 (at risk)	13	(54%)	0.70	0.20 (0.41–1.2)
13+ (high risk, referral to specialist)	21	(71%)	1.00	0.99 (0.74–1.35)
Data from relationship form				
Afraid of current/previous partner or someone important to you				
No	162	(56%)	1.00	
Yes	34	(65%)	1.10	0.51 (0.83–1.45)

*Based on final retention status (as at March 2018)

^#^Percentages differ from overall retention rates due to missing data on some characteristics

### The rate of home visit completion and delivery of programme content

The extent of completion of home visits (mean number and actual vs. scheduled) is reported in Table 5. This analysis is based on the 191 women whose completed home visits were known by December 31, 2015. Women who completed the Central Australia FPP to the child’s second birthday had a mean 46 home visits, which is equivalent to nearly 75% of intended home visits. Completion rates were slightly higher during pregnancy (81%) and slightly lower during infancy and toddlerhood. For clients who left the programme prior to the child’s second birthday, visit completions were close to 60% for the period the women were in the programme. For those still active as at December 31, 2015, the number of home visits and completion rates were similar to those graduating from the programme.

**Table 5:** Mean number of completed home visits and actual vs. scheduled visits (%) by programme stage

Group*	Mean number actual visits			Actual vs. scheduled visits (%)		
	Pregnancy	0–1 year	1–2 years	Pregnancy	0–1 year	1–2 years
Completed programme (to child’s second birthday)	9.1	20.2	17.0	81%	69%	72%
Left programme during						
Pregnancy	2.6			31%		
Infancy	6.7	6.6		62%	23%	
Toddlerhood	7.8	15.9	5.1	65%	55%	22%

*For the 238 clients (80%) with complete home visit information as at December 31, 2015

The mean time spent on each broad programme area and estimated percentage of planned content covered during home visits by programme stage (group) are presented in Table 6. Across all programme stages (pregnancy, infancy and toddlerhood), nurses spent on average over 70 min per client visit. During pregnancy, the most time was spent on *personal health* (32%) followed by *maternal role* (22%). During the infancy and toddler stage, *maternal role* was the content area on which NHVs spent most time, followed by *personal health*. NHVs reported covering more than 80% of programme content across their visits.

**Table 6:** Mean time spent on each programme content area (minutes and percent) and the percentage of planned content covered

Group*	Mean time, min (%) spent on each programme content						Mean total time per visit (min)	Percentage of planned content covered (%)
	Personal health	Environmental health	Life course development	Maternal role	Family/friend relationship	Health/human services		
Completed programme to child’s second birthday								
Pregnancy	24 (32%)	8 (11%)	8 (11%)	18 (23%)	10 (13%)	7 (10%)	76	86.3
Infancy	14 (17%)	9 (12%)	10 (13%)	27 (34%)	10 (13%)	9 (11%)	79	82.5
Toddlerhood	12 (16%)	10 (12%)	13 (16%)	26 (34%)	11 (14%)	6 (8%)	78	84.0
Left program								
Pregnancy	25 (29%)	9 (11%)	10 (12%)	18 (22%)	13 (16%)	9 (11%)	84	86.8
Infancy	14 (18%)	10 (12%)	11 (14%)	25 (32%)	11 (14%)	9 (11%)	79	83.0
Toddlerhood	13 (16%)	11 (14%)	13 (17%)	24 (32%)	10 (13%)	6 (8%)	77	85.6

*For the 238 FPP women with complete home visit information as at December 31, 2015

## Discussion

To the best of our knowledge, this is the first time (a modified version of) the Olds’ Nurse Family Partnership model has been delivered to an Australian Aboriginal cohort (or other First Nations Peoples) in a remote region and the acceptability and feasibility of implementation reported. Over a period of nearly 7 years, the Central Australia FPP has reached over 40% of eligible clients. The 60% not taking up the programme reflect a combination of failure to refer, offer of referral which was not taken up or a formal referral which was declined. Studies tend not to report programme reach, so this statistic cannot be compared with other settings.

Women who were not referred to the programme appear to be less vulnerable than those referred, and those accepting were younger and had less stable housing than those who were not referred or declined participation. Of women who were referred and offered a place, only 27% declined to participate, mostly older women and those with three or more children, who are often ineligible in other settings. Even with the broader eligibility, the rate of those declining the programme is similar to that reported for other home visiting programmes in the USA, including the NFP, at around 25% (Gomby et al. 1999; McGuigan and Gasssner 2016).

The Central Australia FPP achieved a similar retention beyond the child’s first birthday and until programme completion at the child’s second birthday (53 and 41% respectively) to that reported for community delivery of the NFP in the USA (51 and 40% respectively) (O'Brien et al. 2012). Given the high mobility of Central Australia FPP clients, evidenced by the large percentage of clients moving out of the service area and house moves data, this level of retention could be viewed as a considerable achievement for the Central Australia FPP model. Attrition before the child’s first birthday due to “addressable” reasons was lower for the Central Australia FPP than for the US community delivery of the NFP (O'Brien et al. 2012) (59 and 80% respectively). This mainly reflects a lower proportion of women leaving the Central Australia FPP due to “unable to locate” (2.3 vs. 13.9%) or “Excessive missed appointments/attempted visits” (18.6 vs. 36.3%), (Central Australia FPP and the US NFP respectively). The local community knowledge of the ACWs, the major adaptation of the FPP to Aboriginal Australia, is considered critical in this outcome. Non-addressable reasons for attrition were higher in the Central Australia FPP than for the US NFP, “clients moving out of the area” (O'Brien et al. 2012) (34 vs. 13%). Looking at possible ways to continue to deliver the programme to women who move out of the service area is a matter for programme development.

Compared to the US NFP, the Central Australia FPP achieved a greater number of completed home visits (mean 45 visits per client compared with 33 for the US NFP) and a greater proportion of completed visits relative to the number intended (75% in the Central Australia FPP compared to 55% in the US NFP). The data for both studies relate to women who were still enrolled or had completed the programme (Gomby et al. 1999). A completion rate of 75% of intended visits is also considerably higher than other home visiting models, such as Hawaii’s Healthy Start (42% of intended visits) and the Parent As Teacher programme (56% of intended visits) (Gomby et al. 1999). In addition, the Central Australia FPP delivered 80+% of planned programme content during home visits, an important step towards achieving the expected outcomes of the programme (Gomby et al. 1999).

The current study provides evidence that it is feasible to implement a modified NFP model in an Aboriginal community in Central Australia which is acceptable to young Aboriginal women, as indicated by high programme uptake, good rates of retention, level of home visits and delivery of programme content. This outcome is exceptional given the Central Australia FPP is being delivered in a highly challenging setting in terms of remoteness, transience of the client population who typically fail to engage with services, low levels of education and literacy and complex household structures. It is also a very different cultural setting to that for which the programme was initially devised.

A number of factors are likely to have contributed to successful implementation of the programme. It is implemented by an Aboriginal community-controlled health service which is the major provider of primary health care services for Aboriginal people in the Alice Springs area, and of maternal and child health services for Aboriginal women, which was also the main source of referrals (Wilson 2009). Having provided culturally appropriate antenatal and postnatal care to Aboriginal women (Ah Chee et al. 2001) for many years, the health service is well-accepted by the local communities, and this long-established relationship will have facilitated on-going contact by the NHVs and ACWs.

Employing and training ACWs to work alongside the NHVs have been an important adaptation. ACWs assist in recruiting and retaining women in the programme, in part by helping to locate families during frequent moves. The low proportion of clients leaving the programme due to “unable to locate” or “excessive missed appointments/attempted visits” is likely a reflection of the ACW involvement.

The health service is considering a possible expansion to the role of ACWs to increase their involvement in the home visits and in facilitating access to other needed services. More generally, as a mature programme, with comprehensive information about programme participants, there is now an opportunity to explore possible refinements to enhance retention and programme relevance (Zarnowiecki et al. 2018).

Given the challenges in implementing health care interventions for Aboriginal populations in remote settings, it is encouraging to observe high rates (relative to comparable programmes) of programme reach, retention, rate of home visit completion and delivery of programme content. Our analysis would suggest that the Central Australia FPP provides a platform for improving the health of highly vulnerable women and children. It will be important to continue to monitor community engagement and evaluate programme outcomes. The ultimate test of success is the achievement of programme objectives of improvements in child health and development, reduction in child abuse and neglect and improvements in women’s pregnancy outcomes and parental life course (https://www.anfpp.com.au/).

### Study Limitations

Programme reach may be overestimated if not all pregnancy outcomes in the target population are captured in the clinical database of the health service. However, any adjustment is likely small. In 2005, a leading Australian demographer concluded that “The Main Clinic [of the Central Australia Aboriginal health service] apparently provides a primary care service to almost all indigenous residents of the Alice Springs Aboriginal & Torres Strait Islander Commission Region (with the exception of some older children and teenager males)” (Condon 2005).

While drawing on a reasonably wide set of attributes, the analyses of predictors for retention in the programme might have excluded other factors that could affect client retention. For example, we did not explore characteristics of the programme and staff (such as staff turnover).

We note that our analysis of retention was focused on the child’s first birthday; however, in the absence of an outcome analysis, it is not certain if this cut point is critical.

## Conclusion

Our study provides strong evidence that a modified NFP model can be successfully implemented in a remote Aboriginal population in Australia. The distinct features of the implementing agency likely play an important role in the positive implementation outcomes. While the implementation outcomes are encouraging, it is important to closely monitor and evaluate programme outcomes.

The first step for a successful programme is successful implementation. It seems clear that the Central Australia FPP does provide a vehicle for engaging vulnerable Aboriginal women during the crucial period of pregnancy and their child’s earliest years, with the prospect of better health and wellbeing in mother and child. If the expected outcomes are realised, a narrowing of the health gap between Aboriginal and Torres Strait Islanders and the rest of the nation is a possibility.

## Footnotes

1 In this paper, the term Aboriginal refers to all Aboriginal and Torres Strait Islander people.
